# “Make it possible for more people to work at home!” representations of employee motivation and job satisfaction in Danish and Norwegian newspapers during the COVID-19 pandemic

**DOI:** 10.3389/fpsyg.2022.972562

**Published:** 2022-09-12

**Authors:** Katrine Sonnenschein, Øivind Hagen, Ingrid Steen Rostad, Ragnhild Wiik

**Affiliations:** ^1^Department of Leadership and Organizational Behaviour, BI Norwegian Business School, Trondheim, Norway; ^2^Department of Leadership and Organizational Behaviour, BI Norwegian Business School, Stavanger, Norway

**Keywords:** remote work, COVID-19, employee motivation, employee satisfaction, digital workplace

## Abstract

During the COVID-19 pandemic, many employees with task-based jobs were forced to work from home, while others were furloughed or laid off. The current study aims to investigate how Norwegian and Danish newspapers represent employee motivation and job satisfaction of remote workers in light of the COVID-19 pandemic. The study used a thematic analysis of five newspapers from Norway and Denmark with different daily distributions and political orientations. The findings suggest that the newspapers in the two countries represented the topic of interest from different perspectives, and this led to the use of two motivation theories: the self-determination theory (SDT) and Herzberg’s two-factor theory. The SDT helps us understand why some employees feel motivated and are more productive while working from home. The need for autonomy, competence, and connectedness is being satisfied for some employees but not for all, which may affect the strength of employees’ job motivation. Herzberg’s theory helps explain physical and psychological issues as dissatisfiers, as these issues are the consequence of working in a home-based office. Furthermore, a hybrid model seems to be an optimal solution for the future job market, where employees with task-based jobs can feel motivated and job satisfied while working either from home or from the workplace. Finally, it is important for employers to look after both the physical and the psychosocial conditions if hybrid solutions are going to replace the traditional workplace.

## Introduction

Being work-life researchers, we found that the closing down of the economy during the COVID-19 pandemic represented a historically unique opportunity to study systematic and widespread changes in work-life balance. In particular, we were interested in how companies adjust to a completely new situation and how remote workers’ job motivation and job satisfaction develop under these new conditions. As such, the pandemic and the measures taken to deal with it represent a unique laboratory from a work-life research point of view (Kniffin et al., 2021). The focus of the present paper is on how Danish and Norwegian newspapers discuss the motivation and job satisfaction of remote workers during the COVID-19 lockdown from 11 March 2020 to 31 July 2021 (at the time of the data gathering the lockdown was still in effect).

COVID-19 is a pandemic with comprehensive economic consequences internationally, as industries around the world have been forced to shut down to prevent the spread of the virus. This situation is a serious challenge for employees, as they have had to work from home or were furloughed or laid off. Other employees in life-sustaining positions such as medical staff had to work under often difficult conditions (Kniffin et al., 2021). The need for employees to work from home due to the COVID-19 pandemic has been facilitated by the ground-breaking development of communication technologies (Kniffin et al., 2021). The emphasis in the current paper is related to employees with task-based jobs. The job market researcher Steensgaard (2020), referred to in the article by Svendsen (2020), argues that those with task-based jobs are flexible in relation to time and place and constitute most of the remote workers during the pandemic, in contrast to those who deliver services and need to be present at their workplace. The concept of “remote work” includes “work from anywhere” (i.e., not necessarily at home) (Kniffin et al., 2021). However, in the current article, “remote work” and “remote workers” relate to those working from home only due to the pandemic. Newspaper coverage of the topic reflects and affects society and changes in society by reporting, discussing, and analyzing what happens on a daily and immediate basis. Unlike scientific scholars making analyses based on distance in time, theory and stringent scientific methods, journalists report instantly, with fewer filters and procedures. Newspapers provide raw and less processed data and are therefore an adequate source of documentation for the current study.

The two Scandinavian countries (Denmark and Norway) that have been chosen as a case have some qualities that make them interesting for a study on work-life. Scandinavia is a geographical region with a common culture and linguistic similarities. The work-life in the region is considered pioneering on several factors related to sustainability, egalitarianism, industrial democracy, equal opportunities between men and women, and a comprehensive welfare and social security system (Strand and Freeman, 2015; Hvid and Falkum, 2018). The three economies (Norway, Denmark, and Sweden) are furthermore characterized by a shift away from an industrial-based economy toward a more service- and knowledge-oriented economy and a high degree of digitalization (Eriksen, 2016). The Economist (2013) has described the Scandinavian economic system as the “the next supermodel,” focusing on the mix between market orientation and state interference when evaluating which regions tackled the financial crisis of 2008/9 best. So far, it appears that the Scandinavian countries–particularly Norway and Denmark–have dealt well with the pandemic, measured in death tolls and the degree to which the economy shut down. Explaining factors are said to be high levels of trust, well-functioning social safety systems, equality, high housing standards, strong digital infrastructure, high testing rate, etc. (Erikstad, 2021; Kielgast, 2021). Compared to Norway and Denmark, fewer businesses in Sweden closed down in the period investigated (Hale et al., 2021).

Furthermore, the reason for focusing the present study on the Scandinavian region is due to a lack of academic literature on how remote workers’ job motivation and job satisfaction developed under the COVID-19 pandemic. We have identified only a few European based studies which investigate the wellbeing of employees during the pandemic (Bentall et al., 2020; Van Der Feltz-Cornelis et al., 2020; Gorgenyi-Hegyes et al., 2021; Mihalache and Mihalache, 2022; Sischka et al., 2022). These studies discuss how the COVID-19 pandemic impacted the physical and mental health of employees in various sectors and how organizations can alleviate the negative effects of the COVID-19 pandemic by providing support to employees. However, only one Scandinavian study on the motivation and job satisfaction has been identified (Da et al., 2022). The study undertaken on Norwegian employees found that conflicts in the family due to working from home was detrimental to the employees’ wellbeing. In the opposite direction, support from leaders, colleagues, and family, presented a positive impact on their wellbeing. All the studies identified were quantitative and none of them based their empirical data on media. For these reasons a qualitative study based on newspaper articles from Denmark and Norway on the given topic seems timely.

### Research question

The following research question assisted us in investigating the topic of interest of the current paper:

*How do Norwegian and Danish newspapers represent employee motivation and job satisfaction of remote workers in light of the COVID-19 pandemic?*

In the next section we describe the qualitative methodology that we applied to answer our research question.

## Theory

We will use the self-determination theory (SDT) and Herzberg’s two-factor theory on motivation and hygiene (Herzberg et al., 1993; Deci and Ryan, 2000, 2008) as our theoretical framework. According to Ryan and Deci (2000), the antecedents of job motivation are based on the satisfaction of three basic needs, which are “innate, essential, and universal” (p. 232). These needs are autonomy (feeling of having the opportunity to make own choices), competence (feeling able to accomplish something), and relatedness (feeling of belonging to someone). The levels of experiencing autonomy, competence, and relatedness will predict the intrinsic motivation toward a particular task (Breaugh et al., 2018). The strong and positive relation between satisfaction of basic needs and motivation is also emphasized by Maslach and Banks (2017). Breaugh et al. (2018) argue that employees who have all the basic needs satisfied are “inherently satisfied by their work itself or because they have identified with its purpose. These feelings create a strong sense of job satisfaction” (p. 6). Job satisfaction is defined as the positive attitudes, judgments, and feelings toward work tasks.

Herzberg distinguishes between dissatisfiers (hygiene factors) and motivators (motivation factors). They are not associated with the work itself but with factors that surround the doing of the work. These dissatisfiers include working conditions, salary, company policy and administration, benefits, job security, supervision, and interpersonal relations (Herzberg, 1959). When these factors are below acceptable levels, job dissatisfaction occurs and thereby motivation and productivity are inhibited (Herzberg, 1959; Herzberg et al., 1993). This is in accordance with the theory of Maslow (1987), who states that satisfaction of physical environmental needs clears the way for performing at higher levels. According to Herzberg (1959), motivators are achievement, recognition, the work itself, responsibility, and advancement. Interestingly, according to Gorgenyi-Hegyes et al. (2021), physical and mental health “internal locus of control factors” are self-managed by employees during the COVID-19 pandemic and hence these factors lead to their wellbeing, but do not lead to workplace satisfaction. This is due to the fact that employees worked remotely and felt responsible themselves for their own emotional and physical wellbeing. On the contrary, facilities that are provided by the organization “external locus of control,” i.e., insurances and preventive care led to satisfaction with their workplace and were thus considered hygiene factors (Gorgenyi-Hegyes et al., 2021).

Different from Herzberg’s two-factor theory, the SDT does not include dissatisfiers such as inappropriate working conditions, which may explain the dissatisfaction of employees when their needs regarding physical and psychosocial working conditions are not being met. In this regard, the two theories complement each other, and they will both be applied in the current study to explain the job satisfaction and motivation of remote workers in light of the COVID-19 pandemic.

Productivity, i.e., the output per worker, is almost everything in the long run (Krugman, 1994). Pritchard (1992) defined productivity as the balance between the costs of doing business and the value of the outputs. These two definitions are in harmony with the traditional one that states that productivity is the ratio of output over input (Hannula, 2002; Coelli et al., 2005; Mandl et al., 2008; Wiik, 2011; Roghanian et al., 2012). Another definition is that productivity is the combination of efficiency (doing things right) and effectiveness (doing the right things) (Mandl et al., 2008; Roghanian et al., 2012). Regardless of the definition, productivity will in one way or another be influenced by both physical and psychosocial working conditions.

## Methodology

### Data collection and data

To obtain a breadth of perspectives, we chose five newspapers from Denmark and Norway (in the Danish and Norwegian languages) with different political orientations and daily distributions. Dagens Næringsliv (96,000), Politiken (227,000), and Berlingske Tidende (145,000) are large distributions. Klassekampen (Monday–Friday: 34,000; Saturday: 53,000) and Information (79,000) are smaller distributions (Index Danmark/Gallup, 2022; Mediebedriftenes Landsforening, 2022). The various political profiles of the newspapers are shown in Table 1 (see table below) (Hjarvard, 2007; Klassekampen, 2016).

**TABLE 1:** Characteristics of the data sources and data material.

Paper	Paper profile	Time of collection	Data base	Total no. of articles	No. of articles after selection
Dagens Næringsliv	Neo-liberal	June/July 2021	Subscription based digital archive	239	19
Klassekampen	Left	June/July 2021	Atekst/Retriever Norge		17
Politiken	Social-liberal	June/July 2021	Info.media	356	8
Berlingske	Conservative	June/July 2021	Info.media		15
Information	Independent	June/July 2021	Info.media		2
Total				595	61

The newspaper articles were identified in the period from 1 January 2020 to 31 July 2021. The two countries shut down for the first time on 11 March 2020 (Denmark) and 12 March 2020 (Norway), and they experimented with different degrees of re-openings during this period.

Through an inductive approach, we used combinations of the following search words when looking for relevant articles in the databases: “COVID”/“corona” (and “korona” in Norway as the word is written with both “c” and “k”), “pandemi” (“pandemic”), “COVID,” “arbeid,” “arbejde” (“work”), “digitalt arbeid,” “digitalt arbejde” (“digital work”), and “hjemmekontor” (“home office”/“working from home”). The following databases were used to search for newspaper articles: Atekst, Info.media and a subscription-based digital archive. Atekst and Info.media were available through Danish and Norwegian libraries. The newspaper articles referenced here can be found in the above-mentioned databases by searching the articles by title.

The first round resulted in 595 articles in total. Of these, only 61 newspaper articles met our criteria. These criteria were:

1. Articles discussed advantages and disadvantages of home working due to the COVID-19 pandemic in a Scandinavian context.
2. Articles discussed home working in relation to task-based jobs in a Scandinavian context.
3. Articles referred to the points of view of workers, managers, and other professionals in a Scandinavian context.

This relatively small proportion of qualifying articles is due to the fact that many of the articles that included the term “work,” “pandemic,” “COVID/corona” had topics that are not relevant to this paper’s subject matter (e.g., the workload of those who deliver services and who are thus not performing task-based jobs).

### Data analysis

We were inspired by the thematic analysis approach (Braun and Clarke, 2006, 2019) in that we identified themes inductively. First, we used open coding where we labeled meaningful units of the text independently. Codes such as “home office provides stress relief,” “less time spent commuting,” and “home office allows more time for children and housework” were created. In the next step, we organized the various open codes into categories which then were generated into the four following themes: “Health-realted issues with the home office”; “More autonomy when working from home”; “Forced digitalization”; “Working life has changed forever, the home office is here to stay” (Braun and Clarke, 2006). The point of departure for our exploratory approach was to find out how Norwegian and Danish newspapers represented the digital workplace of task-based jobs during the 2020–2021 pandemic lockdown period. Nevertheless, through our thematic analysis it appeared that the focus of the newspapers was on employees’ motivation and job satisfaction in the new work setting, which prompted us to narrow our research question.

To ensure the validity of our approach, all the authors engaged in the data analysis (e.g., investigator triangulation) (Denzin, 1978). Three of the authors were responsible for coding articles of different newspapers divided amongst them. One author was responsible for the first open coding of Berlingske, Politiken, and Information. Another author was responsible for the coding of Dagens Næringsliv and a third author coded Klassekampen newspapers. Each author reviewed the coding of the other authors to ensure consistency in the coding process. Finally, the authors agreed on the broader themes. Table 2 demonstrates an example of the organization of codes and categories based on the theme “More autonomy when working from home.”

**TABLE 2:** Example of open codes, categories, and theme.

Open codes	Categories	Theme
*More flexibility at the future* *workplace* *Less time spent commuting* *Home office allows more time for* *children and housework* *Many employees appreciate* *flexibility* *Home office provides stress relief* *Home office leads to more* *productivity* *Less controlled management leads* *to increased employee wellbeing* *Survey shows that people working* *from home perform better*	Positive outcomes of more autonomy at the workplace	More autonomy when working from home
*Forced home working can lead to demotivation and less autonomy Self-management requires* *discipline* *Self-management may be a* *challenge* *Difficult to find a balance between* *work and free time when working* *at home* *The Norwegian labor law was* *breached* *Work-life balance is difficult for* *women to maintain*	Negative outcomes of more autonomy at the workplace	

## Findings

In the analysis, four themes were generated as most relevant for covering the newspapers’ conceptualization of employee motivation and job satisfaction of remote workers during the pandemic (More autonomy when working from home; Health-related issues with the home office; Forced digitalization; Working life has changed forever, the home office is here to stay). The newspapers contained a variety of perspectives including those from professionals (psychologists, medical doctors, physiotherapists, chiropractors, and lawyers), consultants, unionists, employers, employees, journalists, and researchers. The employers and employees worked in various sectors: media; banking; real estate; teaching; health; ministries; consultancies, large private companies (such as telecommunication and oil companies); and pension funds.

### Health-related issues with the home office

#### Physical health issues

Several articles discuss health-related issues with respect to working from home. To summarize, when at home, employees often work at a coffee table or other unsuitable places. The consequences of working in badly equipped home offices are back pain, lower back, and neck pain, as well as headaches. Furthermore, unhealthy working routines that such as eating lunch while working and failing to distinguish between work and leisure time may impact negatively on their wellbeing (Kehlet, 2020; Kjær and Olsen, 2020). Kjær and Olsen (2020) explain:

“Everything from bedside tables to dining tables have been used as home workplaces, and it is unfortunately often combined with unhealthy work rhythms where work time never really ceases–and where people see themselves run to the fridge, quickly make themselves some food, then work on while eating.”

Despite these health-related issues, most Danish employers seem reluctant to reimburse their employees for any equipment purchased to work at home. Their argument is that the employees already save time and money due to the lack of a commute. Furthermore, economic assistance with homework equipment may have some tax-related consequences for employees since it is regarded as part of the salary (Gourani, 2020). Finally, Danish companies normally do not assist their employees with moving work equipment from the office to their home (Kjær and Olsen, 2020).

Musculoskeletal pains are the most frequent reason for sick leave in Norway. About 32% of the staff at the telecommunication company Telenor have reported more physical pains than usual when working from home during the pandemic (Sollien, 2020). Employers are urged to take responsibility for their employees’ physical wellbeing when working at home. In an interview, a lawyer specialized in work-related issues explained the following:

“Employers who in the first hectic months before the summer were busy handling new digital programs and finding routines for follow-up and meetings must now take a far greater responsibility for how the employees actually feel in the home office (Mikkelsen Espeland and Birk Tjeldflaat, 2020).”

#### Mental health issues

Not only may physical health issues occur due to working at home, but the general wellbeing of employees is also considered a problem. For example, depression, anxiety, stress, and loneliness are normal phenomena amongst people working from home (Kaspersen, 2020; Kehlet, 2020). Interestingly, one article mentions that mental health-related issues may also occur upon return to the workplace due to the readaptation process, which can be challenging for some people, and counseling may be needed (Skarum, 2021). Other articles agree that employees will experience a loss of quality of life by being back in the office every day and suggest a hybrid solution to satisfy the needs of employees (Hessel, 2020; Kehlet, 2020; Poleszynski, 2020; Skarum, 2021; Steensgaard, 2021a,b):

“Many prefer that home working or so-called hybrid working, where you work both at home and in the office, becomes the new normal (Skarum, 2021).”

“In my opinion, there are therefore good reasons to expand schemes that make it possible for more people to work at home–whether they want to be at home one, two, three, or more days a week (Poleszynski, 2020).”

### More autonomy when working from home

Some newspapers discuss the positive outcomes of employee autonomy while working from home: less time spent commuting (an advantage for both employees and society due to less time spent in transit and less stress); working from home when children were sick; housework which could be performed during work hours. Furthermore, working from home might have provided considerable stress relief (Mikkelsen Espeland and Birk Tjeldflaat, 2020; Hilstrøm, 2020a; Poleszynski, 2020; Steensgaard, 2020, 2021a; Rogaczewski, 2021).

Contrary to what many employers had expected, working from home did not make employees less productive. Some articles even argue that employees have been more productive when working from home during the pandemic (Hessel, 2020; Steensgaard, 2021a). An article on the World Economic Forum’s website in August 2020 stated that digital homework during the pandemic has “ushered in a new era of productivity, inclusiveness and connectedness” (Hessel, 2020). One explanation for the increase in employee productivity when working at home is managers’ inability to exert full control, which gives employees the autonomy and motivation to perform their best at work (Grinde, 2020b; Zacho Haarde, 2020a,b).

While working from home can be advantageous for some, autonomy can be a challenge for others. Home schooling children and the temptation to do housework during breaks or instead of working can present challenges (Ejsin, 2020; Hilstrøm, 2020b; Steensgaard, 2021a). Being forced to work from home can also affect employees’ autonomy negatively and make them feel demotivated (Kopperud et al., 2020). Some employees have succeeded in structuring their home office workday, however, others found the distinction between work and leisure challenging, as described in Dagens Næringsliv (Grinde, 2020b; Kaspersen, 2020):

“We are logged in around the clock and an excuse about poor coverage and lack of access is simply no longer valid. It can mean both demands or a feeling of always having to be available, and then the distinction between work and leisure becomes unclear, she says (Mikkelsen Espeland and Birk Tjeldflaat, 2020).”

Interestingly, Norwegian labor law was breached by many employees due to their flexible working hours, as they frequently worked late. According to the law, employees have the right to a minimum of 11 h of uninterrupted free time (Finstad and Bugge, 2020). Women in particular may have difficulty finding a balance between work and life when the job moves home. An article entitled “Career Break for Women” (Flatum and Gjøvikli, 2020) states the following:

“Many experience that the distinction between work and leisure is erased and the balance disappears. Women who work a lot find it particularly difficult to maintain a ‘work-life balance’.”

For many, a lack of balance also threatens career development. With home offices, men and women tend to go into traditional roles, where women take on a larger share of the housework than men, which affects job performance and career development possibilities. One article refers to the English Financial Times, which asks whether the pandemic is about to take women back to the 1950s (Paulsen, 2020). In another article written by two female managers in a consulting firm, home working is described as the home office trap, as women are encouraged to come to the physical office to pursue career opportunities (Flatum and Gjøvikli, 2020).

### Forced digitalization

Digitalization is another recurring theme. One of the newspaper’s commentators discusses how the pandemic has led to the realization of the digitalization that we have heard could be possible in working life since the beginning of the 2000s, but which has not occurred until now. The pandemic has thus been a breaking point for a process of change. She says it in this way:

“It is also possible to see it as if a pandemic were needed to finally speed up the Wonderful New Working Life, which has been announced on behalf of the creative classes since the 90s: with independence and solo work and home working and digital meeting activities with and without kids appearing on the screen. Now we get to try it out (Grinde, 2020a).”

Other articles also focus on how the pandemic has led to a long-promised and positive digitalization, and that forced digitalization will give us a different familiarity with the technology from what we had before. An interviewee in an article says it like this:

“I am absolutely certain that the corona crisis opens up possibilities for more familiarity with digital aids, which we can enjoy even after this is over (Zacho Haarde, 2020b).”

The same interviewees talk about how, for example, video conferencing equipment has been boosted by the pandemic:

“We have had the opportunities and the equipment for a long time, yes. The process has just gone so terribly slow. When you are an overworked General practitioner (GP), experiments with video consultation are not what you jump at first (Zacho Haarde, 2020b).”

The interviewee describes here how the pandemic has forced actors who had previously been too busy to experiment with more efficient technological solutions to do just that.

In an article, the British sociologist Giddens is interviewed. He is talking about a “Digidemic” rather than a pandemic. He looks beyond the pandemic and is more concerned with the enormous change that digitalization is contributing to:

“In my eyes, man has already merged with the machines. Just look at the relationship we’ve got with our phone. This small machine, which is made on a technology that alone is 40 times stronger than the technology that made the moon landing possible 50 years ago (Zacho Haarde, 2021).”

The Giddens interview represents one of the few exceptions where the newspaper takes a step back and concerns itself with something other than immediate problem-solving and handling the pandemic.

### Working life has changed forever, the home office is here to stay

Several of the newspaper articles present working from home as a game changer for working life as we knew it. One article from Berlingske says that “2020 was goodbye to working life as we knew it” (Hilstrøm, 2020a). There is no turning back to the old habits of going into the office every day. It seems that home working gained status during the pandemic. While before the pandemic many managers regarded working from home as a way of getting away from work demands, it is now recognized that efficiency and productivity are not reduced when employees work from home. In an interview in Berlingske, the director of the international company Experian says that:

“The corona period has taught us … that it is quite possible that a flexible working life in the home office leads to efficiency. So something good has come out of the corona (Steensgaard, 2021a).”

A specialist psychologist and associate professor, interviewed in Dagens Næringsliv about the future use of home working, points out: “The problem is not that you become less efficient at working from home, but that you become more limitless’ (Kaspersen, 2020).”

This article represents one of the few critical views/perspectives in the newspapers on the practice of home working. More frequently, the use of a home office is represented as a positive opportunity for employees to exercise more flexibility and autonomy at work. The articles also demonstrate the workforce’s general interest in working more from home after the pandemic. One article explains:

“There is no doubt that the experience of home working will have an impact on how the physical working life of knowledge workers in particular will take shape in the future. The big question, however, is what it will look like in practice (Steensgaard, 2021a).”

It is important that managers take into consideration not only the individual needs but also the company’s needs as a whole. The balance between physical and digital work must thus be discussed between managers and their employees. In some cases employees can work from home and in other cases they need to be physically present. For example, in the creative stages of projects, the physical presence of employees is important (Mikkelsen Espeland and Birk Tjeldflaat, 2020; Steensgaard, 2020). However, some companies may give their employees full freedom to choose their place of work (Ejsin, 2020; Gourani, 2020; Kjær and Olsen, 2020; Lynklip Svansø, 2020; Otto, 2020; Rubin, 2020; Steensgaard, 2020). Danske Bank is an example of a company giving its employees full freedom to choose where they want to work. For those employees who choose to work from home, 8,000 DKK will be provided for equipment for their home office. More digital collaboration does not necessarily mean that colleagues will have more superficial relationships with one another. Digital collaboration may be combined with more events where colleagues actually meet and socialize at work (Rogaczewski, 2021). Such events will be a way to create a connection among employees, develop cohesion, and foster an organizational culture (Lynklip Svansø, 2020). Rogaczewski (2021) explains:

“More digital collaboration does not necessarily mean a more superficial relationship with each other… But only if more digital collaboration is combined with greater, better and more intense social contact, when we are actually together physically, will the two things complement and support each other.”

## Discussion

Despite the themes identified in the newspapers being separable, they are also intertwined. We discuss them within the perspectives of health and intrinsic motivation and digitalization.

### Health-related issues with the home office

Reznik et al. (2021) agree with the statements in the newspaper articles identified in this study that remote work can have an impact on the physical health of employees in various ways, including “a lack of exercise, over-eating, musculoskeletal issues, and pain” (p. 1). Many employees use kitchen and dining tables, dining chairs and bedrooms, which are not ideal for posture and can cause ergonomic problems. During the pandemic, employees often exercised less and ate more while working from home. The reasons were, among others, the lack of a commute between home and the workplace and less access to fitness and sport centers during lockdown. Furthermore, working at home made access to food much easier than at the workplace (Reznik et al., 2021).

According to Davis and Kotowski (2015), employers will need to provide safe home-based offices for their employees to prevent musculoskeletal disorders. Increased cases of musculoskeletal issues among employees will have a serious impact on companies’ income due to treatment and compensation costs and absenteeism. Employees can also prevent musculoskeletal disorders themselves by taking regular breaks, e.g., every 20–30 min (Davis and Kotowski, 2015). Furthermore, approximately 1 h of exercise outside of the home every day is recommended. Finally, meal planning and using a room other than the kitchen will prevent excessive eating (Reznik et al., 2021).

The academic literature also agrees with the newspaper articles identified in the present study that mental wellbeing has worsened for many employees who have been working from home during the pandemic. For example, COVID-19 has led to employees experiencing stress/burnout due to the changed working conditions and exposure to COVID-19 news in the media (Kniffin et al., 2021). Furthermore, employees may experience techno stress due to the use of new information technologies and the work addiction that makes them unable to disconnect from work-related ICT (computer, phone, etc.) outside of normal work hours (Brivio et al., 2018; Mun et al., 2022).

The loss of social connections among colleagues due to working from home may lead to loneliness. Social interactions—both formal and informal conversations among colleagues—are important for the wellbeing of employees (Mogilner et al., 2018). Virtual communications may lead to misunderstandings among colleagues and thus to feelings of rejection and loneliness (Cacioppo et al., 2006). Since the lockdowns in spring 2020, depression and anxiety have spiked among the population (Bentall et al., 2020). According to Şentürk et al. (2021), “poor sleep quality, trouble focusing at work, being female, workplace loneliness, low levels of control over working hours, and low levels of physical activity were predictors of depression. Poor sleep quality, increased workload, and being female were predictors of anxiety” (p. 41). Employers need to address employees’ mental wellbeing in general in their Human resources (HR) practices. It is also important to offer a culture in which employees feel comfortable discussing how they are doing. For example, initiatives such as virtual lunch meetings, information about working from home, access to counseling, therapy, training, etc. can be provided. Research on such initiatives may even inform future practices to improve employees’ psychological wellbeing (Kniffin et al., 2021).

Like one of the newspaper articles in the current study (Skarum, 2021), Hamouche (2020) discusses the need for organizations to develop a special plan for employees returning to the workplace after having worked remotely during lockdown to avoid stress and mental health issues. Employers should also discuss with their employees future expectations and the company plan before their return to the office. Such initiatives may prevent adaptation problems after their return, as described in some of the articles in the present study.

Concerning working from home, the newspapers emphasize employees’ physical and mental health problems such as musculoskeletal issues, difficulty concentrating at the home office, feelings of depression, anxiety, techno-stress, loneliness, and insomnia. Such problems may be caused by unsatisfactory hygiene factors which inhibit motivation and job satisfaction. Other newspapers argue in the apparent opposite direction, i.e., that motivation and job satisfaction have increased during the pandemic. This is due to satisfaction of needs related to other hygiene factors (work flexibility and a better work-life balance) (Herzberg et al., 1993; Holmberg et al., 2018). The apparently contradictory views on satisfaction of hygiene needs on motivation and job satisfaction may be explained by need variations among employees and variations in home office conditions. Also, the results of Gorgenyi-Hegyes et al. (2021), may explain the apparent contradictions. Since physical and mental health is considered within the employee’s internal locus of control and thereby not hygiene factors which is the employer’s responsibility, such issues may not necessarily be highlighted when it comes to motivation and job satisfaction.

Regardless of different views, working from home seems to have systematic influences on health, motivation, and job satisfaction.

### Intrinsic motivation and digitalization–the way forward

The newspapers identified in the present study discuss the outcomes of employees’ increased autonomy while working at home during lockdown. The SDT helps explain this phenomenon. When an individual has autonomy, he or she is not controlled by external forces such as reward and/or punishment (Deci and Ryan, 2008; De Charms, 2013). Furthermore, SDT defines the need for competence as the need to master the environment and develop new skills. Finally, the need for relatedness represents the need to feel connected to others and to see themselves as a member of a group (Van den Broeck et al., 2016).

The newspapers in this study demonstrate that employees feel more autonomy and are more productive than before due to working from home. Aligned with the SDT, this phenomenon can be explained by a lack of full control by managers, which makes the employees feel more autonomy and thus leads to better performance (Deci and Ryan, 2008). However, other employees may feel forced to work from home against their will, which negatively affects their feeling of autonomy and makes them less intrinsically motivated. The lack of autonomy may be felt particularly among women, who are still doing most of the housework during the pandemic, which affects their career development. Indeed, research demonstrates that some employees working from home during lockdown have reported greater difficulties completing their work when they had to do home schooling and child care compared to those without children at home (Yang et al., 2021).

Competence as described by the SDT is also discussed in the newspapers in this study. Some articles argue that the increased use of digitalization enables employees to gain more familiarity with the technology, e.g., through the use of different applications which may enhance their competences, although some employees considered this process to be slow and tedious. Employees may feel stress when there are increased requirements in information technology which exceed their capacity in terms of the level of difficulty or the amount of work (Tarafdar and Gordon, 2007). The increased use of technology can also lead to less relatedness among colleagues, which according to the SDT may cause less intrinsic motivation and less wellbeing.

However, some newspaper articles report that digital collaboration does not necessarily mean that collegial relationships are more superficial, as they may be combined with regular physical social events to strengthen the organizational culture and cohesion. Moreover, various newspaper articles discuss the importance of a post-pandemic hybrid workplace to ensure that employees are also physically present at the workplace and not just virtually connected. Yang et al. (2021) agree that the hybrid workplace can be introduced after the pandemic, and that future research needs to determine the ideal number of days working at home vs. in the office; the level of employee autonomy to decide their own workplace flexibility; and the types of tasks that are more effectively undertaken at home vs. at the workplace. For example, according to two newspaper articles (Mikkelsen Espeland and Birk Tjeldflaat, 2020; Steensgaard, 2020), during the creative stages of projects employees should be present to ensure the quality of the work. Allen et al. (2015) agree with this argument, stating that teammates who interact remotely miss the creative benefits that can flow from face-to-face meetings.

The three basic needs to achieve intrinsic motivation and job satisfaction (autonomy, competence, and relatedness) seemed to be achieved by employees to some extent during the pandemic. Some employees felt more autonomy due to the lack of control by their manager. Furthermore, their technological competence increased to a large extent. However, being forced to work from home caused less motivation among others and the lack of relatedness among colleagues caused mental health issues, such as feelings of loneliness, depression, anxiety, etc. After the pandemic, the basic need for relatedness will be ensured again through a hybrid workplace, where the combination of working from home and in the office will be made possible at many workplaces.

## Limitations

The limitation of the study included relatively few number of newspapers of the two countries. To have a more complete picture of the representation in Danish and Norwegian newspapers of employee motivation and job satisfaction of remote workers in light of the COVID-19 pandemic, more newspapers with large distributions could have been included, such as the Norwegian newspaper “Aftenposten” and the Danish newspaper “Jyllandsposten.” Furthermore, local newspapers could also have been included in our study. However, by including both big and small newspapers with different political orientations we have strived to undertake a representative study of the newspapers’ coverage of our topic of interest.

The data analysis of the present study may have been subject to a certain level of bias. As researchers at the same university, we were all required to work from home during the lockdown and we may thus have interpreted data in a way that was close to our own experiences. However, through the investigator triangulation where we cross-checked each other’s coding and thematic analysis, we aimed to prevent such bias.

## Conclusion

Our findings showed that the newspapers in the two countries analyzed the motivation and job satisfaction of employees working from home during the pandemic from different angles, which led us to use two motivation theories to discuss the phenomena: the SDT and Herzberg’s two-factor theory. The SDT helps us understand why some employees indeed feel intrinsically motivated and are more productive while working from home. The data demonstrates that the need for autonomy, competence, and connectedness is being satisfied for some employees but not for all, which may affect the strength of employees’ job motivation. Herzberg’s theory helps explain musculoskeletal issues and feelings of depression, anxiety, stress, loneliness, and insomnia as dissatisfiers, as these issues are the consequence of working in a home-based office. However, the data also demonstrates that motivation and job satisfaction have increased during the pandemic due to the satisfaction of needs related to other hygiene factors (work flexibility and a better work-life balance). It is important to recognize that people are individuals and not a homogenous group, and therefore there is not an optimal type of workplace that fits all. A hybrid model seems to be an optimal solution for the future job market, where employees with task-based jobs can feel motivation and job satisfaction working either from home or at the workplace. When working from home, it is important for employers to look after both the physical and psychosocial conditions. In particular, this needs to be emphasized if home working or hybrid solutions are going to replace the traditional workplace on a large scale.

We analyzed newspaper articles from both big and small newspapers with different political orientations. The newspapers contained the voices of managers and employees from a broad spectrum of the job market–both private and public companies. In addition to managers and employees, other experts were interviewed (psychologists, medical doctors, physiotherapists, chiropractors, and lawyers). These diverse perspectives represented in the newspaper articles led to some generalization of the findings in the Norwegian and Danish context. However, further research needs to be done to have a complete picture of the Scandinavian newspapers’ representation of employee motivation and job satisfaction of remote workers in light of the COVID-19 pandemic. In this regard, a larger number of Norwegian and Danish newspapers as well as Swedish newspapers need to be included in future studies.

## Data availability statement

The raw data supporting the conclusions of this article will be made available by the authors, without undue reservation.

## Author contributions

KS contributed with the conception and design of the study, methodology, theory, data analysis, discussion, and conclusion. ØH and IR contributed to the conception and design of the study, data analysis, and methodology. RW contributed to the theoretical section, discussion, and conclusion. All authors contributed to manuscript revision, read, and approved the submitted version.
